# Epidemic Influences, and the Duties of the Medical Profession in Relation to Them

**Published:** 1851-04

**Authors:** Theodore S. Bell

**Affiliations:** Louisville, Ky.


WESTERN JOURNAL

OF

MEDICINE AND SURGERY.

APRIL, 1851.

ART. I.—*Epidemic influences, and the duties of the Medical Profession in relation to them.* By THEODORE S. BELL, M. D., of Louisville, Ky.

It is a matter of gratulation that in all civilized regions at this time, a number of leading medical minds are engaged in the all important duty of watching the phenomena attendant upon epidemics, and of recording facts upon the subject. At no period of medical history has there been more devotion to the highest interests of medical science, than at present, and the greatest results among the rich acquisitions of modern medicine, are found in the domain of public hygiene. Hygiene is one of the highest departments of medical philosophy, and he is totally unworthy to be a member of any branch of the medical profession, who is unacquainted with the characteristics facts, and principles of modern hygiene. The ancients regarded a knowledge of the causes of things a source of peculiar felicity, but the physician who is ignorant of the causes of those matters that concern the health or life of those who confide in him is an evil to himself and to the community. The pressure of the public suffering, the devastations of disease, and the consequent injury to the public prosperity, have, in Europe, forced upon the attention of the medical profession the demands of public hygiene. Large masses of gleaners are abroad upon the fields of disease, gathering facts, and enforcing truths upon the minds of the rulers of the land. Chairs have been established in the medical schools of portions of Europe, the occupants of which are devoted to the cause of the public health. The political rulers of England, France, Austria, Prussia and Russia have shown a commendable zeal in this great work, and have clearly recognised the great and important truth, that the protection of the health of the community is a duty of Government. The reports of the Registrar-General of England are invaluable in showing the sources of endemics, and the ease with which they may be removed. A great deal of labor has been bestowed upon the sources of disease in England; and a most commendable zeal has been displayed, in the construction of a system of public hygiene, that is honorable alike to English medicine, and to the cause of humanity. The medical periodicals of Great Britain teem with publications on this high interest, and abundant proof is accumulating to show that the mind of the profession is properly directed and is engaged in the pursuits that belong to it, and that are conducive to the good of the people.

The position of American public hygiene, we regret to say, is not of that exalted character that it should have attained. There is but little zeal, devotion or care on the subject, and communities are permitted to live in open defiance of sanitary laws, and to build up, and chain sources of pestilence, lest some accident should cut them adrift. Many years ago, Dr. Rush proclaimed, in his usual eloquent and energetic style, the great truths: "*to*

*all natural evil, the author of Nature has kindly prepared an antidote. Pestilential fevers furnish no exception to this remark. The means of preventing them are as much under the power of human reason and industry, as the means of preventing the evils of lightning and common fire.*" American physicians entered upon the course indicated by Dr. Rush, and in portions of the country their labors were efficient and successful. Many eminent teachers and writers had definite ideas of the cause of fever, and of the means of preventing them, and they exerted themselves in the work of prevention. But that the principles of public hygiene were not generally understood, nor promoted, is abundantly proven by the history of epidemic cholera in the United States. Instead of gathering facts in order to see whether that disease might not be an old enemy in a new disguise, the idea was at once adopted that epidemic cholera was a new disease the cause of which was unknown, and could not be traced. The wildest hypotheses sprang up in the midst of the bewildering phases of the epidemic, and any conjecture, however untenable, was deemed more trustworthy than the soberest truths. The contest seemed, for awhile, to be, to try who could stray farthest from the paths of the inductive philosophy, and, it must be confessed, that many were remarkably successful in their efforts. If the great mass of American physicians had studied the habitudes of the great pestilence, from 1832 to the present time, in the only way they should be studied, the profession would occupy a much higher position on the subject, than it now commands. Facts have accumulated abundantly, on the cause of cholera; they speak but one language, and that is clear and conclusive. These facts are of no dubious import, no matter whether they are gathered on the banks of the Hoogley, amidst the brackish pools of Kastan, the passes of the Balkan, the sewers and cess-pools of London, in the wet and filthy squares of Louisville, or upon the decks of Western steamboats.

The season for Epidemic Cholera is approaching, and it is a matter of public duty to arouse attention to the necessity of preventive measures. Even with all the information that has grown under the influence of carefully recorded observation, even in the presence of the most incontestable proofs of the value of that information in its striking and successful results, it is lamentable to think how much remains to be done, in the improvement of the sanitary condition of large sections of the country, and how incessantly the medical writer who recognises his duty, is compelled to urge the claims of health upon the public attention. Even when he succeeds in awakening attention to the demands of the subject, unceasing vigilance is necessary, lest the attempts at improvement may be so badly managed as to increase the very evil they were intended to prevent. The claims of the subject are large, the labors required to meet them are great, but in the discharge of the duties of public hygiene, no labor, that is essential to success, can be too great. The truths of that science are a gospel for the corporeal part of man, and, for that, are what the Apostle Paul said he was—a savor of life to life to those who believe, and of death to death to those who reject the truth. It is appalling to look over the earth, even in the best regulated communities, and see every where the most shocking disregard to the plainest and most palpable dictates of ordinary reason and common sense in matters concerning the public health. It is dreadful to look upon communities standing upon the verge of disaster, where a change in the impalpable matters of breath, over which changes men have no dominion, may convert health into disease, and disease into death. When we read of the sword of the Syracusan tyrant, suspended over the head of Damocles, by a hair, we naturally conclude that we should have hastened from the unhealthy region; but do we not see communities sleeping quietly under threatening dangers suspended by a tenure quite as precarious as that which held the sword over the head of Damocles?

The duty of the public hygienist then is clear and indisputable. As long as the necessities for his truths exist, he must press them home upon the public. He must purify the air by the lustrations of his facts, and build up confidence in his mission, by the successful results of his labors. In the performance of this duty, I shall take the habitudes of cholera, as illustrations of the hygiene truths, which it is the purpose of these remarks to enforce.

In 1832 I first became acquainted with cholera, and it roused in me a very sincere desire to understand it thoroughly. In the works of Senac, Torti, and Morton, I had read of diseases precisely analogous to this, but the latter was called a new pestilence. Senac called his cases Malignant intermittents, Torti and Morton called theirs, Pernicious fevers. In comparing the accounts they gave of those diseases, with what I saw in the disease called cholera, it was palpable to me that there was not an essential feature of the disease known as Pernicious fever that was not present in cholera, and after a very patient and thorough analysis of the whole subject, in perfect conformity to those rules of philosophising which Bacon and Newton announced, and which all experience has confirmed as true principles, I felt persuaded that epidemic cholera was the product of that combination of solar heat, moisture and dead vegetable matter, which has been made familiar to medical men by Lancisi. This conclusion was not rashly made; the responsibility of announcing this result was well weighed, and under a due sense of that responsibility, I declared the truth in the Transylvania Medical Journal for 1832. At that time the medical mind, nearly all over the world, was bewildered as to the cause of the pestilence. Error as to the cause was rife and baleful; the ignorance that repudiated all knowledge of the cause was scarcely less baleful. It was difficult then, as it is now to imagine how men could shut their eyes to the facts that uttered no uncertain sound, and which spake no dubious language. It was evident then that medical men preferred talking of the mysterious character of what had no mystery about it, rather than make a bold and studied effort to remove the mystery. A distinguished contemporary has uttered a truth of no ordinary import in the following language: "in medicine, loose and imperfect statements are little less hurtful and misleading than positively false ones." Who has not often felt the force of this truth in his search after medical truth? Let us impress this matter by a notable and recent instance in our own country. The Philadelphia Board of Health, in their report of 1849, use the following language: "the appearance of the cholera at Staten Island (New York,) and its almost simultaneous outbreak at New Orleans, is one of those peculiar coincidences *which will forever be shrouded in obscurity.* Carried into both ports by emigrant ships from Havre, where when they sailed there was no cholera known to exist—the one leaving on the 3d of November, and the other on the 9th, following nearly the same track, the disease appearing at sea on the 25th November, in one, and on the 28th in the other, *when most probably in the vicinity of each other*, crowded with emigrants, uncleanly and badly ventilated. The inference is that they must have passed through a stratum of atmosphere loaded with some peculiar influence which, under favorable circumstances, produced in both cases the cholera poison." There is reasoning with a vengeance—the appearance of cholera at Staten Island and New Orleans, almost simultaneously is one of those coincidences that will forever be shrouded in obscurity, but the coincidence of the attack of two ships, which are guessed to have been, at the time, in the vicinity of each other, admits of an easy inference—the Philadelphia Board of Health rush to a conclusion with railroad celerity—"the ships must have passed through a stratum of atmosphere loaded with some peculiar influence," etc. Now, why consign one of the coincidences to a never ending obscurity, instead of guessing through it, as in the other case? Would it not have been quite as easy to guess that a stratum of atmosphere loaded with pernicious influences settled down precisely on Staten Island and upon New Orleans, about the same time, as to manufacture this stray waif of atmospheric poison for the two ships at sea, which might not have been in the vicinity of each other? Is it any wonder that the public hesitate in granting confidence to such extraordinary reasoning, such miserable guesses, such palpable absurdities as those contained in the extract quoted from the Report of the Philadelphia Board of Health? Would it not be wonderful if the Board itself, had any faith in their own precious and marvellous philosophy? And is it from such experience, such philosophy, such wild and inexcusable dreaming, as that of the Philadelphia Board of Health, that a sound and reliable system of sanitary truths can spring? If so, it must be hoped in a system of compensation—as they started a stratum of air loaded with cholera poison, to attack the two emigrant ships, at sea, they may have strayed away from their poisonous waif, towards the truth, and reached it by accident. The quotation made from the Report of the Philadelphia Board of Health is an excellent illustration of the truth quoted from the British Foreign and Medico-Chirurgical Review,—"in medicine loose and imperfect statements are little less hurtful and misleading than positively false ones."

Let the light of truth now look into that awful shroud of never-ending obscurity, of which the Board speaks, and see whether the mystery is so very impenetrable. I

pray the reader's very particular attention to the facts, which high medical authority declares are shrouded in total obscurity, The case before us is full of instruction of the highest importance, the facts are clear and indisputable, and if the inferences deducible from them are not palpable, it would be scarcely worth while for the profession of medicine to have facts—they are useless, if they cannot be used.

The statement of the Philadelphia Board of Health that there is a profound mystery in the fact that two ships should have had cholera upon them, in the midst of the Atlantic Ocean, when the port from which they had sailed was free from the disease, and nothing of its existence was known at any of the points from which the emigrating passengers had started. Now there is no more mystery in these facts than there is in an outbreak of yellow fever, or any other disease that springs from a local cause on board of a ship, and medical history is filled with examples of this kind. The very fact that numerous other ships were sailing in the same regions that were traversed by the two cholera ships, and escaped a visitation of the pestilence, should open the eyes of medical philosophy to the possibility that the cause may have been local, and the conditions for its production must have been confined to the ships that exhibited the effects. There is certainly nothing irrational in this view, nor is there anything illogical in this train of reasoning. How a waif of cholera atmosphere could have struck these two ships alone, and at a distance from each other, and permitted all others to escape, is a point of serious enquiry, especially as we not only have no evidence that a cholera atmosphere can travel across water, but the evidence is conclusive that it cannot. And then there are facts that show that the cause of cholera on board one of the ships under review, the New York, was confined in its operations, and that it could not have lain perdu in the atmosphere, until it dropped down upon the devoted ship. *Not one of the cabin passengers on the ship had the disease.* We are required to believe that the passengers of one of the infected ships inoculated the citizens of New Orleans with cholera, and caused an awful epidemic, but in doing so we must shut our eyes to the fact that the cholera victims of the packet New York could not inoculate the cabin passengers confined to the same vessel. But we cannot reach truthful conclusions, by shutting out facts—the whole subject must be surveyed, and carefully analysed.

But let us dispose of the case of the packet ship, New York. She arrived at Staten Island, on the 2nd of Dec. 1848, and had had cholera aboard for the space of seven days, among the emigrant passengers in the steerage. The remaining steerage passengers were placed in quarantine, at Staten Island, in the public stores on the Dock. Numbers broke the quarantine, and escaped into the city. In a crowded and filthy German boarding-house, two fatal cases occurred—the place was at once cleansed and purified, and the occupants, two hundred in number, were scattered over the city. In a German boarding-house of a more respectable character, a case occurred, which could not be traced to any medium of connection with the emigrants on the packet ship New York. One of the supposed emigrants went to a building accupied as a convalescent hospital for typhus patients. He did not communicate the disease to any of the inmates of the hospital. "This," says Dr. Buel, to whose paper on the subject I am indebted for these facts, "it is believed, terminated the first brief chapter in the cholera of 1848." A sharp frost closed its operations in the city of New York, in December, 1848, for Dr. Buel says, that the weather in that city, though mild and temperate, was wintry.

But let us now turn our attention to New Orleans, and see if local causes have anything to do with cholera. Dr.

Fenton dates the appearance of cholera, in New Orleans, in 1848, on the 11th of December, and makes it coincident with the arrival of the ship "Swanton." We shall look at this point directly. The ship "Swanton" had two hundred and eighty steerage passengers, consisting of German and French emigrants. If the presence of cholera in New Orleans was due to infection from the ship "Swanton," we must find, upon clear and indisputable evidence, that there was nothing like cholera at New Orleans, antecedent to the arrival of the vessel, and we must also have an approximation to the state of things in New York, under the infliction of an analogous cause. A ship arriving in New York with small-pox among the passengers would produce very much the same occurrences in New York that would take place in New Orleans, under a similar arrival.

"For several weeks previous to the arrival of the 'Swanton,'" says Dr. Fenton, "the weather had been changeable, *for the most part very warm,* though there had been several white frosts. Yellow fever had almost disappeared, and there was but little sickness prevailing; though, *among the existing diseases, were observed some remarkable cases of stomach and bowel complaints.* On the 5th of December, (six days before the arrival of the "Swanton,") I attended a gentleman on Custom-house street, who labored under vomiting, pains and spasms in the bowels; and prostration to such a degree, that, if epidemic cholera had been supposed to be here, no person would have hesitated to pronounce him a case." [Are we to infer from this that it is necessary that numbers of cases shall occur, in order to decide the nature of an individual case?] "He had no rice-water evacuations, his bowels were rather costive, and he vomited bile; but many such cases have been seen since the epidemic was declared."

"Some days previous to this, three or four negroes were attacked with cholera morbus on the same night, in

Gravier street—they were promptly treated, and all soon recovered, similar cases were observed in the practice of a number of physicians in different parts of the city, all going to show, as it appears to me, that the *epidemic influence of cholera was gradually being matured in our midst.*"

The ship "Guttenberg," from Hamburg, where cholera was prevailing, arrived at New York, five days before the "Swanton." Cholera appeared among the passengers of the "Guttenberg," before she left the Elbe, but as soon as the vessel got out to sea, the disease subsided completely and no more cases occurred during the whole voyage.* There is certainly nothing unreasonable in the presumption that, as cholera was prevailing at Hamburg, at the time, and the passengers of the "Guttenberg" got on the vessel at that city, and all the cases occurred before she got out of the Elbe, the cause was local, and originated at Hamburg. After getting out to sea, the disease subsided completely, showing there was no local cause on the ship. How she managed to escape that wandering waif of cholera atmosphere so poetically imagined by the Philadelphia Board of Health, and so accurately located in the neighborhood of Nova Scotia, we are at a loss to imagine. She was in that region about the time the packets "New York" and "Swanton" were.

But let us return to the "Swanton," one of the ships attacked by the stray waif of the Philadelphia Board of Health. This vessel was out from Havre twenty-six days before a death occurred, the first being from *consumption*, on the 28th of November. After this sixteen or seventeen deaths occurred during the passage, most of them from bowel complaints, *supposed to be from dysentery.* If they were supposed to be from dysentery, it is self-

---

* [Fenton's] Southern Reports.

i.e. Fenner evident they could not have been from cholera, for none but an idiot could confound the two diseases.

On the day after the arrival of the "Swanton," at New Orleans, a woman was carried from the ship to the Charity Hospital, and found to be in a complete state of collapse. It was at once decided to be a case of Asiatic cholera. On the following day, another case from the ship, was carried to the hospital, in a complete state of collapse. *Three other cases of cholera, from different parts of the city, and not of the ship*, were admitted to the hospital, on the day this case entered. On the evening of the same day, a gentleman having business near the St. Charles Hotel, called on Dr. Fenton, with strong symptoms of cholera. He had not been near the ship Swanton, nor had he seen any of her passengers.

On the 15th of December, there were eight cases of cholera admitted into the Charity Hospital, and Dr. Fenton heard of cases in the private practice of a number of physicians. The intelligent reader cannot fail to see the material differences between the results in New York and New Orleans, four days after the arrival of the vessels at the respective ports. And can there be any difficulty in ascertaining whether there were differences between the two cities in any particulars connected with the production of disease, that may account for the differences in the results? Is it not given to the reason of medical men to find a solution for problems of this kind? Has medical experience accumulated no certainties on the subject of the cause of disease? Does eternal night reign over medical facts alone? Do the changes of seasons affect vegetable life, and produce no effects on animal life and animal health? These are questions of momentous importance to every medical man, and should be carefully studied, analysed and directed in their channels. I shall consider them directly—the facts reported by Drs. Buel and Fenton are reported so clearly and faithfully, that they are perfectly reliable. As an evidence of the care necessary in considering facts, the following specimen, selected from a large number of reputed cholera facts, is recorded by Dr. Fenton. It is very expressive.

A learned physician of New Orleans, addressed a letter to a distinguished Professor in Paris, which was published in one of the New Orleans papers, in which the writer mentioned three fatal cases of cholera, that occurred on Custom-house, Bienville, and Chartres streets, on the 15th of December. The writer adds: "It is well enough to remark here, that these three primary victims of cholera in New Orleans were all cooks, going every morning, very early, to the principal market in the city, situated on the bank of the river, a cable's length from the infected vessel." These are not only "loose and imperfect statements," but "positively false ones." The ship Swanton, from which these statements were intended to show the New Orleans cases of cholera were derived, instead of lying a cable's length from the principal market, was *at the upper end of the Second Municipality*, nearly a mile from the principal market frequented by the cooks. It is by the manufacture of facts in this way, that truth is hidden, and falsehood sustained, and if there is any one earthly thing, a thing merely earthly, in which truth is a priceless gem, it surely is in the causes that produce epidemic diseases. If we cannot at all times ascertain the causes satisfactorily, it is not difficult in any case to ascertain the accompanying phenomena. Even these are of some value, because partial knowledge is better than complete ignorance. If all men could agree upon the facts of cholera, a system of sanitary regulations could be adopted that would afford security against the ravages of the disease. It "is the pestilence that walketh in darkness" that takes communities by surprise, not the one that walks in light.

The facts already detailed constitute a starting point for investigation, and before passing to the accumulation of any more, it will be well enough to pause and see those before us. What are they? On the 5th of December, a vessel with cholera on board, arrived at New York; the passengers being quarantined, the disease continues to prevail among them. Numbers break the quarantine and escape into the city—two of these die with cholera—one in a crowded German lodging-house, another in a convalescent fever hospital in the suburbs. Two other fatal cases occur, and immediately after they occurred, a sharp frost comes, and there was no more cholera in New York for months. Those who had been exposed to the local cause took the disease and died—there was no local cause scattered about New York, and the disease did not spread over New York—it is, therefore, abundantly evident that the existence of cholera on board of a ship, is not sufficient to produce cholera among the inhabitants of the port at which she lands, for if such a cause were equal to the production of such effects, the city of New York should have been visited by cholera, in December, 1848. This palpable truth should always be remembered in considering facts that have the *appearance* of militating against this view. The same cause, under the same conditions, will always produce the same effects. We shall have plain sailing if we stand by established principles, and make a wide survey of the facts.

Let us now look at the New Orleans facts. We find that weeks before the arrival of the ship Swanton there was a predisposition in New Orleans to disorders of the bowels—Dr. Fenton says, that "among the existing diseases were observed *some remarkable cases of stomach and bowel complaints*," and Dr. Fenton attended a case with symptoms so nearly resembling cholera, that if that "epidemic had been supposed to be in New Orleans, there would have been no hesitation in pronouncing this a case of that disease." In truth it was a sporadic case of cholera, and this shows that a cause of cholera was in operation before the Swanton reached that port. Here then is a marked and striking difference between New Orleans and New York, and there was an essential difference in another particular—Dr. Buel says that the weather in New York, though mild and temperate, was wintry—Dr. Fenton says: "the weather in New Orleans was changeable, *for the most part very warm*, though there had been several white frosts." The explanation is palpable: there had been weather warm enough to produce the remote cause of cholera, but frost enough to check, modify and, to some extent, control an outbreak of the disease, and all that was needed was *uninterrupted* warm weather to produce epidemic cholera, in the winter months. In New York, not only the months were wintry, but the weather was also, and cholera did not spread, but in New Orleans, a widely varient state of things existed.

Some days previous to Dr. Fenton's case on the 5th of December, three or four negroes *were attacked in the night* with cholera morbus—similar cases were observed in the practice of a number of physicians in different parts of the city, all going to show, as Dr. Fenton very philosophically and logically says: "that the epidemic influence of cholera was gradually being matured and developed in our midst." Dr. Fenton speaks like a philosopher in that sentence. How different the conduct of those who look for the cause of disease in an imaginary stray waif floating in the atmosphere of the ocean, or to some distant land, or in an assumed infected air in a newly arrived ship, instead of using their common sense upon the palpable truths around them.

In New Orleans the weather increased in warmth, and epidemic cholera gained ground. From the 16th of December to the 22nd the weather was "*oppressively warm*," the thermometer rising, according to Dr. Fenton, to 84°.

On the 23d of December the Board of Health announced that cholera was epidemic in New Orleans; it raged severely from the 22nd to the 30th, there being *ninety-two* deaths on the 28th. Citizens of Louisville who were in New Orleans between the 16th of December and the 22nd, say that the weather was so hot that the air was stifling in its effects. From the 22nd to the 30th the weather was cool, wet and gloomy, thus making a general exciting cause for the development of the effects of the remote cause which was acting during the hot weather. From the 12th of December, 1848, to the 20th January, 1849, there were nearly fourteen hundred deaths by cholera, in New Orleans.

We have thus ascertained that as the temperature increased the cause of cholera increased, and the scale for judging this fact, is the safe one found in the effects. If the cause was dependent upon the warmth of the weather, parity of reasoning would show that cold weather would check the cause. Did this actually occur? That faithful observer, Dr. Fenton, says it did. He states: "On the night of the 30th there fell a white frost. On the morning of the 1st of January, there was another white frost, and from that time, the disease steadily declined." While the epidemic lasted, its influence was almost universal—nearly every person in the city felt it to some extent.

These facts, then, press home valuable truths to the mind of the intelligent reader. The cause of cholera was not introduced into New Orleans by the ship Swanton, for it was in operation there before that vessel arrived, about the time indeed that the errant atmosphere of cholera was said to be dropping upon the Swanton in the neighborhood of Nova-Scotia. But if we admit the fact that the Swanton introduced cholera into New Orleans, we remove no difficulty; we unshroud no obscurity. The question recurs upon our fancied gain—why did not the packet ship New York introduce the disease into the city of New York? Why were there only four deaths in that city, and fourteen hundred in New Orleans? It is plain, in the presence of such questions as these, that we gain nothing by the admission that New Orleans was infected by the Swanton. We make no advance, by ridding ourselves of one set of meshes, and becoming involved quite as deeply in that of another. The man who steps out of one miry place into another does not improve his condition.

But proofs accumulate in establishing the local origin of cholera. We have seen that the disease could make no headway in New York, during the cold weather, and that during oppressively warm weather in December, in New Orleans, it made a frightful headway. While cold weather lasted in New York, the disease was a nonentity, but as soon as warm weather began to act in conjunction with moisture and decayed filth, cholera began in New York as it had done, in similar circumstances in New Orleans, in the previous December. On the 11th of May, 1849, cases began to occur in the vicinity of the "Five Points." The filthy tenements occupied by the victims, beggar description, and Dr. Buel says: "the small area unbuilt upon is covered with black pools of filthy water." Dr. Buel adds: "at my first visit, on the 16th of May, five human beings, one man and four women, lay upon the floor, in different stages of cholera. There was nothing under them but mud and filth, and nothing over them, but a few rags of the filthiest condition." In fourteen days the pestilence had not extended itself more than 100 yards from its origin, nor had it numbered more than about 20 victims. In New Orleans, in 17 days from the commencement of cholera, the deaths amounted to forty-five. As the weather increased in warmth, the disease spread and raged in both cities. From the 5th of December to the 16th of December the temperature gradually increased, and cholera increased slowly; from the

16th to the 22nd of December the weather was oppressively warm in New Orleans, and cholera increased rapidly in its ravages, and spread itself over the city, and on the 28th reached its zenith. In the city of New York the temperature increased gradually, in May, and cholera increased gradually and slowly. Dr. Buel says: "during the first seven days of June the weather became warm, and the disease spread rapidly in all directions. Within this period 70 cases were received into the hospital, being double the whole number admitted during the whole previous time the hospital had been open. At the end of the first week in June, or about twenty-eight days from the appearance of the first case, the pestilence had become pretty extensively and generally diffused over the city. At the end of the third week in July, or about seventy-two days from its first appearance, it had arrived at its "culminating point." In New Orleans the increase of the temperature, after it began to rise, was rapid, and intense, and a corresponding state of things existed in the march of cholera. The disease in New Orleans was checked by a sudden check of the summer temperature. In New York the temperature and disease increased gradually and uniformly. Its decline depending upon the diminution of one of the elements essential to the production of the cause of cholera—moisture, was gradual and uniform, but more rapid than its increase. In New Orleans the element removed was solar heat, and as the removal was sudden, the effect was as sudden as could be expected where many had imbibed the cause without having succumbed to it, up to the time the farther production of the cause was stopped. In New York, the element removed was moisture, and that was removed more slowly than the solar heat was in New Orleans, consequently the cause ceased its operations more gradually. In the production of the cause of cholera, too much moisture plays the same part that too small an amount does.

It is a great misfortune for the world, and not a limited evil to the medical profession, that such faithful observers and truthful chroniclers as Drs. Buel and Fenton have not been more abundant. Under their luminous details, there is no kind of difficulty in tracing the origin and foot prints of epidemic cholera. If such minds had originally surveyed the pestilence, the profession of medicine would not have been groping in darkness amidst such follies as contagion, infection, portability, stray waifs of atmosphere at sea, telluric sources, animalcular origin, ozone *ignes fatui* and the various other bewildering phantasies in which the medical mind has revelled. Medical observers in various parts of the world, in some degree resembling the excellences of Drs. Buel and Fenton, are beginning to abound, and cholera records are becoming as uniform now as they were formerly discordant.

The malarial origin of epidemic cholera, upon which I started almost alone in 1832, can now boast of numbers of powerful advocates. There can be said of it, what cannot be said of any other cause that has been assumed for cholera. It is the only cause that harmonises with the facts; it is the only cause that has given birth to sanitary laws that have been successful in their operation, and it is the only cause that has enabled men to make predictions that have been verified by the events. On this point I shall speak more fully hereafter.

In the early part of last autumn, I consented to prepare a paper on the history of cholera, its cause, &c., for the pages of a quarterly periodical in the hands of a large and popular denomination of Christians. In that article I introduced a notice of some of M. Quetelet's results of the Theory of Probabilities, and I am gratified to find that their value is attracting the attention of other medical writers. Dr. E. M. Pendleton, of Sparta, Ga., in a paper prepared for Dr. Fenner's 2nd volume of Southern Medical Reports, speaks favorably and encouragingly of M. Quetelet's labors, and of their uses in vital statistics. I think the attention of the profession cannot be too earnestly directed to what may be done for the philosophy of medicine, by a rigid and discreet use of the Theory of Probabilities, and I here republish my remarks, made in the October No. of the Quarterly Review of the Methodist Episcopal Church.

Those who have paid much attentlon to the exact sciences are familiar with the rapid growth of the department of calculation known as the Theory of Probabilities, applied to moral and political matters. This calculus owes its origin to Pascal; but in his hands the conception was crude, compared to the form it assumed under the amazing powers of Laplace. The results of the unrivalled processes of Laplace, in developing the Theory of Probabilities, have been of the greatest advantage in many departments of statistical knowledge, and are destined to the fulfilment of many hopes and desires in national affairs. Among those who have advanced the subject beyond the boundaries of Laplace, M. Quetelet, Perpetual Secretary of the Royal Academy of Brussels, is conspicuous. In 1835, he published two admirable volumes, entitled, "On Man and the Development of his Faculties," which opened up a mine of investigation that has been productive of the greatest benefit. The wonderful achievements of M. Quetelet, in statistics, that present to the common eye nothing more than a congregation of the merest accidents—a chance-medley without rule or law, but which, under the analytical principles of the calculus, assume regularity, order, and precision, of the most astonishing character, are among the magic creations of the new science. Our first acquaintance with the works of M. Quetelet, commenced in the Edinburgh Medical and Surgical Journal, for 1837; since that time we have watched his progress with great interest, and his great work, entitled, "*Lettres à* S. A. R. Le Duc, *règnant*

*de Saxe-Cobourg et Gotha sur la Thèorie des probabilités applique aux science Morales et Politiques,*" is one of the profoundest interest to all who study society, either in detail or under generalization.

There are two principles or laws of Probabilitiy, which were first demonstrated by Bernouilli, and they may give the general reader some idea of the nature of the subject. The first, according to the Edinburgh Review, is:—

"That in any vast number of trials, there is a demonstrably greater probability that the events will happen in numbers proportional to *their respective chances in a single trial*, than in any other *specified* proportion; and secondly, that a number of trials may always be assigned so great, as to make the probability of the events happening in numbers, falling within any assigned limits of deviation from that proportion, however narrow, approach to certainty as nearly as we please."

It is impossible to explain, within the confined limits of this essay, even the principles of the laws of Probability; but though debarred from showing their admirable working, a glance at a few of the results may serve to show the accuracy of the calculus, and its important bearing upon society. If we look upon individual examples of marriage, of the proportion of births of legitimate and illegitimate children, and every thing of that kind, we should be led to infer that the returns of various years would show great varieties in the proportions. But listen to the Edinburgh Review, upon what M. Quetelet has shown by his calculus. That Quarterly says:—

"As instances might be cited, the relative proportion in the births of the sexes already spoken of; the ratio of illegitimate to legitimate births in the same country, and the same section of the population; nay, even the number of the still-born (with a distinct per centage for town and country,) which M. Quetelet has ascertained to be so uniform in Belgium, that, on a total number of nearly 6000 annual cases, the yearly deviation of the mean falls short of 140; the ratio of marriages to the whole population, of second marriages to the whole number of annual marriages, and still more minutely, of widowers with widows, widows with bachelors, and widowers with spinsters; the relative ages of parties intermarrying; and innumerable other particulars; all which, free as air in individual cases, seem to be regulated with a precision, where masses are concerned, clearly proving the existence of relations among the acting causes so determinate, that there is evidently nothing but the intricacy of their mode of action to prevent their being subjected to exact calculation, and tested by appeal to fact. Taken in the mass, and in reference both to the physical and moral laws of his existence, the boasted freedom of man disappears; and hardly an action of his life can be named which usages, conventions, and the stern necessities of his being, do not appear to enjoin on him as inevitable, rather than to leave to the free determination of his choice."

The uninitiated are scarcely prepared for such revelations as these. They are truly astonishing, but they are the mere A. B. C. of M. Quetelet's developments; and we are convinced that even in the hands of that great expounder, the Laws of Probability are yet in their infancy. The future will exhibit their extraordinary revelations in husbandry and in medicine. The inflorescence of plants has been determined under the laws promulgated by M. Quetelet, and their fructification, and other phenomena will be, in time. Boussingault and the Abbè Cotte, have announced "that the arrival of a plant at a definite stage of its growth, is solely dependent on the *total amount* of temperature to which it has been subjected from the first movement of the sap in spring, without regard to its distribution over the intervening time, or the extent of its variations." M. Quetelet proposes, *instead of the sum of the daily mean temperatures, the sum of the squares* of each daily mean, reckoned from the freezing point, "because the force exercised by the temperature is of the same nature as actual force. It is by the sum of the squares of the degrees, not by the simple sum of the degrees, that we must appreciate its action." For instance: "It is found that the common lilac blooms as soon as the sum of the squares, *of the mean daily temperatures*, as indicated by the centigrade thermometer, amounts to 4264°, so that the mean time of its flowering at any given station, may be at once determined from the meteorological records of its climate. *At Brussels, this mean date is the 27th or 28th of April. In other localities, it occurs earlier or later, by about three or four days for every degree of latitude south or north of Brussels, and about five or six days later for every hundred yards of elevation above the elevation of that city, which is itself sixty-five yards above the sea.*"*

But let us return to M. Quetelet:—

"To each plant" (thus he states his general conclusion) "is attached a constant—the square of a certain number of degrees of warmth necessary for the occurrence of 'inflorescence.' Whether a plant is found in such and such a latitude, at such and such a height, in the open air or in a green-house, it is the temperature" (so measured) "that must be considered. Thus are explained all the anomalies that present themselves in this kind of research. Geographical causes have no influence but by the variations they cause in temperature."

We have marked a portion of one of the above quotations in italics, and we pray the reader's strict attention to the facts there detailed. They have an important bearing on the isothermal lines of disease, and will be useful when we reach that part of the subject.

We have dwelt upon the doctrine of the Laws of Probability, not only on account of the intrinsic value of the

---

* Edinburgh Review.

subject, but for the purpose of pointing out its bearing upon medicine. After looking at the results of the calculus in statistical affairs, in their moral and political relations, we should suppose *á priori,* that the Law of Probability could be applied most successfully to medical reports, in all the various departments of medical science. But alas! even the master hand of M. Quetelet starts affrighted at the undertaking. The following remarks of Dr. Holland, of England, in his work on Medical Evidence, impressed themselves on our mind, years ago, and we indulged the confident hope that land was in sight. The Edinburgh Review quotes Dr. Holland as follows:

"A very special advantage has been the application of numerical methods and averages to the history of disease; thereby giving it the same progress and certainty which belong to statistical inquiry on other subjects. Averages may in some sort be termed the mathematics of medical science. The principal is one singularly effectual in obviating the difficulties of evidence already noticed; and the success with which it has been employed of late by many eminent observers, affords assurance of the results that may hereafter be expected from this source. Through medical statistics lies the most secure path into the philosophy of medicine. The inquiries which so greatly distinguish M. Louis, as a pathologist, may be noted as eminent examples of this method, which is now pursued with eminent success by many eminent physicians in our country.'

But M. Quetelet despairingly says:—

"All reasonable men will, I think, agree on this point, that we must inform ourselves by observation, collect well recorded facts, render them rigorously comparable before seeking to discuss them with a view of declaring their relations, and methodically proceeding to the appreciation of causes. Instead of this, what do we see? Observations incomplete, incomparable, suspected, heaped up pell-mell, presented without discernment, or arranged so as to lead to the belief of the fact which it is wished to establish; and nearly always it is neglected to inquire whether the number of observations is sufficient to inspire confidence."

That there is a great deal of truth in the remarks of M. Quetelet, we are not disposed to deny; but there is room also for considerable qualification. The records of medicine are not quite so barren as they are represented to be, nor are they filled with more contradictions than the records of other sciences. Of all the liberal professions, as the phrase goes, medicine is the most thoroughly free. The lawyer is confined by precedents, rules of practice, and time-honored formularies, and he loves the narrow precincts in which he is confined; the clergy feel the restraining influences of the tenets held by the particular party for whom they labor; but in medical science, every man is free to utter such opinions as he may please—those that are valuable are ranked as authorities, those that do not command medical favor and confidence, may find their way into the hands of general readers; and they being indifferent judges, may ascribe great credit to what is entitled to little or none. In this way, such inquirers as M. Quetelet may make grievous mistakes in medical investigations. It is not at all likely that he has studied the works of such men as Louis, Andral, Grisolle, Chomel, Armstrong, and Stokes. If he has studied them profoundly, he has reached a conclusion as to their merits, that varies widely from that of the profession who are qualified to be the best judges. The numerical method of investigating all medical subjects, has done much in its short career, for practical medicine, and it is destined to do much more. The present paths of investigation are leading to grand results. Great names are losing the authority they once possessed, and truth is considered the only master. As a proper corollary to this subject, and an appropriate preliminary to the main question before us, let us briefly examine the positions of Sydenham and Lancisi.

Sydenham was, by common consent, the greatest physician of his age. As a practitioner of the Healing Art, he has rarely been surpassed. He possessed an inquiring mind of a very high order, and when we look at some of his methods of investigation, we are surprised that he left discoveries of the highest importance, to others who possessed scarcely a tythe of his abilities. It is not a little surprising, that with his great reputation, his industry, his careful observation of nature, and his devotion to truth, Sydenham was ignorant of the cause of all the principal diseases of which he treats. Under the veil of a phrase—"an epidemic constitution of the atmosphere"—he worked in darkness. What were the elements of that epidemic constitution; how it was produced; under what peculiar circumstances it showed itself, and by what means it might be prevented, were mysteries to him. He declared that certain disorders, the effect of the "epidemic constitution of the atmosphere," came as regularly every summer, as swallows do, but why they came in summer, he did not know. But while this glorious orb was sinking to rest, a star was rising in Italy, which was preparing to give light to many dark places. As Sydenham was retiring from his labors, Lancisi was qualifying himself for the great work he accomplished, with honor to himself, and benefit to all the succeeding generations of men. In 1717, he revealed to the world the principles of Sydenham's phrase—"the epidemic constitution of the atmosphere"—he conclusively established the elements and mode of its production, the circumstances that created, modified, and destroyed it, and, above all, plainly showed how its noxious influence may be avoided. This was a great step in the progress of medical science, and it may at all times be pointed to, as an evidence of the inestimable boon conferred by medical philosophy upon the human race. But it is lamentable, that the truths of Lancisi's philosophy are not generally familiar to medical men; and that writers on medical subjects often grope in the dark, through their ignorance of what Lancisi taught. The ablest expounder of those views, since the days of the illustrious Italian, is, in our judgment, Professor Caldwell. In not a very limited examination of the medical literature of the past century of years, we have found nothing on the subject of malaria, comparable to the teachings of Professor Caldwell. They are scattered through various treatises that owe their origin to his remarkable abilities, and are worthy the careful study, not only of physicians, but of all who concern themselves with the public welfare. The principles taught by that great master of the subject, are the basis of all the modern sanitary regulations that are founded in reason, and commended by success. The whole subject will pass in review, in the development of the history, progress, and cause of cholera, and to that we turn our attention.

We feel confident that the "Theory of Probabilities," can be as successfully applied to the cause of a large portion of the diseases of mankind as to the phenomena of plants; and if M. Quetelet had investigated the cause of intermittent fever, (and his country presents a rich field for such an investigation,) as he did of the flowering of plants, he would have paved the way, at least, for more important results than any attained in the floral kingdom. There is not, in our minds, any kind of doubt, that the sum of the squares of the mean temperatures of a certain number of days, will as infallibly indicate the production of the malaria that causes a variety of diseases, as it will of the time of the "inflorescence" of plants. For the production of that malaria, certain proportions of moisture, dead vegetable matter, and sun heat, are essential; and if either element is more important than another, it is the sun heat. We know that a certain amount of that, produces intermittent fever; we are constrained to believe that a longer continued term of that element produces graver forms of fever. For instance, yellow fever requires a continuance of tropical heat, for a considerable time, and that disease is mainly confined to what is called the tropical zone. Medical observers throughout the world, have before them a fruitful field for observation. By a careful series of investigations, we may learn to tell with the greatest precision, the approach of various maladies, in regions favorable to their production. Wherever a certain amount of decaying vegetation and moisture is to be found, in the spring of the year, we may take our starting point at the cessation of freezing weather, and find the sums of the squares of the mean temperatures that are essential to the production of intermittent, remittent, and continued fever, cholera morbus, dysentery, cholera spasmodica, with all their attendant trains of kindred diseases. In perfect accordance with that statement, which we marked above, on the effect of degrees of latitude upon the variations of the times of flowering of plants, is every well written record of malarial disease. That there are great variations in seasons, is a well known fact, and the phenomena of nature obey these variations. If the spring is backward, in its usual features, the products of the earth are backward, and malarial diseases are equally affected. If there is unusual summer heat, and an unusual fall of rain in a fertile region, while vegetable life is more than usually active, human maladies are also more abundant. It is by close observation of these natural phenomena, that we are able to foretell a healthy or unhealthy season; and in the ratio of the study of the revelations of nature, is the ability to know her intentions before they are uttered, and this ability is due to the discoveries of Lancisi, which he published in 1717.

In order to make some approach towards an understanding of the character of the agent of sickness, first properly described by Lancisi, I beg leave to submit to the attention of the reader some personal facts that seem to bear strongly upon the cause of, what are called, epidemic influeuces. I hope that no one will so far misapprehend me as to suppose that the statements are made in any spirit of egotism—the sole object is to assist in the establishment of the fact that the cause of any effect must be known, when the most accurate predictions are made as to what that cause will or will not do, and when these predictions are made publicly, in different years, and under circumstances that perilled reputation.

In 1833, an endemic cholera was raging in Lexington with a fatality beyond anything that had ever been known in the West before. As it had attacked a place previously celebrated for its salubrity, fears were entertained that no place was safe from its ravages, and there was some consternation in Louisville. There was some cholera in Louisville, but it was so very limited in its range, that it would have excited but little attention, except for the condition of things at Lexington, and Simpsonville, the latter twenty-two miles distant from Louisville. In the midst of this state of things, I wrote a letter to Professor Yandell, at that time editor of the Transylvania Journal of Medicine, and I affirmed that the range of cholera in Louisville would be confined to the sporadic cases then occurring. The prediction was based upon the fact that previous to the date of the letter there had been no favoring circumstances for the development of a general malaria, and the season was too far advanced for the rains of that stage of the season to do much harm. These were the reasons given in the letter. It was also stated that cholera would be confined to the vicinity of marshes and ponds. If the statements predicted had been postponed until after the disappearance of the disease from Louisville, early in the fall of that year, they could not have been improved in accuracy.

In the winter of 1849, while the cholera was in New York, among the passengers of the packet New York, and while it was raging in New Orleans in the manner described already in this essay; while the steamboats were almost daily arriving at Louisville with cholera cases aboard, who were scattered over various parts of the city, a great deal of alarm was created for the safety of this city. The statement was constantly made that cholera had been introduced into New Orleans by the Swanton, and the expectation that it might be introduced into Louisville by the steamboats from New Orleans, seemed reasonable and well grounded to many persons. In this state of things I thought it due to the public that its fears should be quieted, and that the philosophy of medicine should be vindicated, and in a popular and widely circulated newspaper of the city I announced that the fears of the public were groundless; that no matter how many persons might reach here from New Orleans, with cholera, and no matter where they might be housed, Louisville was in no kind of danger. We were too far advanced into the winter to begin to suffer from the malaria of the previous fall, and had not reached a temperature that could produce the cause of cholera locally. It was also announced that Louisville could have no indigenous cholera until May or June. Each and every one of these predictions was verified to the very letter—numbers of cases were landed here, from New Orleans, in January and February, the boats suffered severely in their upward voyages from New Orleans, but not in the slightest degree in going down from Louisville, and there was not an indigenous case in Louisville, up to the time covered by the prediction. When the design of making these predictions was announced to a medical friend, he earnestly begged me not to hazzard so much upon so variable a matter as cholera, and he was assiduous in endeavoring to deter me from, what he considered, an adventure. But however variable cholera may appear to others, I knew that it had its fixed certainties, and I had unlimited confidence in what I knew of them. A sense of duty weighed down all personal considerations, and the announcement was published. The first victims in 1832, 1833, and 1849 were made in the same locality, and 10 square feet would have covered the space first assailed in the three years mentioned; I knew the conditions that caused those attacks, and I have never known of the occurrence of a case of cholera, without those conditions, and I am as well satisfied that there never was a case without them, as I am of the cause of small-pox.

The third prediction was made under peculiar circumstances. On the 24th of July, 1850, the people of Louisville were aroused by the news that a cholera, unprecedented in its fatality, in Louisville, had displayed itself in the lower end of the city. On the north and south side of Market street, between 10th and 11th streets, in a square not by any means densely populated, and certainly not pre-eminent among the squares of Louisville for its filth, there were fifty cases of cholera, and up to 11 o'clock, on the night of the 24th, there were thirty deaths. The news spread as though it rode upon the electric fluid, and consternation spread with it, even in this community, which is not notorious for its fear of disease. Vast numbers of persons were preparing for flight, and there were few who could boast ease of mind. Worn out as I was, by the performance not only of my usual professional duties, but with my labors as a member of the Board of Health, which labors had been earnestly invoked by the city authorities on the afternoon of the fatal 24th, midnight found me in want of repose. But I greatly feared the result of the facts of the mortality, when they should be made known next morning, and I felt that it would not be right to go to sleep without preparing an antidote, and at 2 o'clock that night, I handed the manuscript of the following article to the night hand of the Daily Journal, and it appeared in that paper on the morning of the 25th:

*The Plague Spot in Louisville.*—The following lines of Byron give a fearful picture of the condition of a small spot in Louisville, if we except the numbers, on Monday night:

> The city lies sleeping,
> The morn to deplore it,
> May dawn on it weeping:
> Sullenly, slowly,
> The black plague flew o'er it—
> Thousands lie lowly;
> Tens of thousands shall perish—
> The living shall fly from
> The sick they should cherish.

The city of Louisville slept on Monday night, little dreaming of the fearful revealing that Tuesday morning was to make. The general health of the city was so far removed from such a state of things as was developed yesterday, that few would have considered it possible for such a reality to burst upon the vision of Louisville. But the pestilence has shown in awful tones the truth of the remark of the Registrar-general of England, quoted a few days since by the Louisville Board of Health: "Cholera is a health inspector that speaks in language which nobody can misunderstand!" If intelligent medical inspectors, armed with less desolating powers than the one who has awakened the attention of the people of Louisville, had made an inspection of the narrow space between Tenth and Eleventh streets, on Market street, this pestilential visitation might have been avoided, if the statements of all medical records can be depended upon. We made a thorough inspection yesterday of the part alluded to, and we could not have believed it possible before, that there was a spot in this city that had boldly defied all sanitary measures, and challenged pestilence to do its worst, in that narrow domain. All measures of hygiene that have done a noble work in other parts of this city were utterly neglected and scorned in this plague spot; yet of all other places we have seen none ever required the fullness of sanitary precautions more. And it devolves upon every man in the city to pay prompt attention to the subject now—we mean to-day. Let no one indulge the foolish dream that a noxious air that rises in the night and sweeps two sides of a street, for the space of an entire square, with the besom of destruction, is to be confined long to the source of its origin. It will gradually creep onward, extending the sphere of its desolation, striking down the strong man, the delicate woman, and the helpless child with equal facility, unless human wisdom and a prompt energy shall take the matter in hand at once. Let not the sun go down on a duty unperformed.

We carefully inspected the region yesterday where the pestilence rioted in its narrow boundaries, and any one who may do the same thing can be at no loss to account for the fact that cholera displayed such powers of havoc on Market street, between Tenth and Eleventh. We will endeavor to describe the region, and hope that we shall be able to make the matter plain without a diagram.

The space alluded to in these remarks was once the site of ponds, and was filled up for a street and for building lots. It is low and flat, with about as dismal a chance for drainage as the genius of pestilence could desire. It is known as "springy ground," against which the essay we published last week warns people as sites for houses. The rains of the spring and summer saturated with water all the dwelling grounds of the space we have descrbed; the old pond bottom holding the water pent up; pools stood under houses; cellars were filled with water—one smoke-house, just above Tenth street, had deep water in it, and in that lot three persons died, and the ground of the infected square was so boggy that a stick pushed into it a short distance would cause water to bubble up. Over this substratum of peril, in some places, were accumulations of filth from 16 to 18 inches deep.

Immediately west of this range, on the north-western side of Market and Eleventh, stands an old pond running diagonally across a square. On the eastern edge of this pond, between it and the plague spot, stands a rope walk, running from Market toward Main street, and between the rope walk and Eleventh street, running parallel with the rope walk, is a strip of earth made by throwing, for some years, the garbage and filth of the streets into the pond. This mass of decaying vegetable filth rested upon water—upon an old pond, the bottom of which holds water as secure as a porcelain wash bowl can, and upon this mass the unusual rains of spring and summer have beaten, until the moisture remained but a few inches below the surface, in full communion with a sunny temperature that almost reached tropical heat. The pond has been kept well filled with water until the last two months, during which time evaporation has gone on with rapidity, exposing a marshy border of some six or seven hundred feet. The shives of hemp were thrown from the hackling house of the rope walk into the pond, and a mass of them that was under water began to show itself in the light of the sun a few days ago, and in a putrid condition. It is difficult to conceive of a more pestilent malaria than that made from this source. While we were making this inspection, the wind was blowing from the west toward the east, inclining slightly to the south, thus blowing the exhalations of malaria from the pond, and directing peculiar violence upon the south side of Market street.

The numerous dwellers around the pond, on the north and south side of Market and the south side of Main street, in the adjoining squares to the seat of pestilence, from whence the wind blew the malaria of the pond, escaped, though much nigher the pond than the victims of the desolating scourge were. We were struck by the fact that a row of houses on the south-west side of Market and Eleventh, which stand some feet below the street, and have filth enough in and around them apparently to breed a pestilence of any given power, have nearly escaped thus far—that row is not in the direction of the winds we have mentioned.

The reader has now before him a pretty full description of the locality of the pestilence—amid its pools of water under the ground floors and cellars of the dwellings, back-yards saturated with water and covered with decaying filth, the marshy edges of the pond, and accumulated scrapings of the streets for years, the cholera sat brooding and hatching a pestilence, and a fearful one it is. Numbers rose yesterday with all the joys of life before them, and before the sun had reached its meridian they were locked in the icy embrace of death—numbers who were waiting upon the dead and the dying after dinner-time, did not live to see the sun-set. In that narrow circle of living beings, there were, up to 11 o'clock last night, fifty cases and thirty deaths—no case has yet recovered, nor shown any signs of recovery. Children that were playing about the floor, upon the entrance of the physician to see other patients, were attacked in his presence and prescribed for immediately, and all have alike died—those in whose cases prescriptions were delayed as well as those prescribed for immediately. The pestilence showed no respect to persons, time, nor medicines. All invocations for relief ended in death. An industrious workman in one of the founderies was summoned from his work, yesterday morning, to see his child—he found it dying—another child was attacked, and he remained with it, and long before night the child and the father were dead. One gentleman came up in town, after breakfast, for a physician to visit his wife, and at 4 o'clock the wife and the husband were dead. These incidents show the fearful power of the pestilence in its confined limits.

We earnestly implore the citizens of Louisville to join the city authorities in measures of self-preservation to-day. Let every survivor in the track of the pestilence be at once removed from the locality, and then let it be thoroughly purified. Let it be stayed without delay. All business should yield precedence to this work—trace the path of the flooding rains in the flat contiguous to the desolated street, and remove the inhabitants, and clean and purify every wet and filthy place. Cover the marshes with sand, and lay it on thick; and the marshy border of every pond should be treated in the same way. Let the work be well and thoroughly done, and make the sanitary condition of the lower end of the town equal to that of the upper end of town. In that district, wherever cholera showed that work was to be done, it was promptly done, and the pestilence ceased. The magnitude of the work requires great efforts, but let each man exert himself, like a man. If we all do our duty, Providence will do the rest. During the day, malaria has but little power; the night is the season of its strength. If we all exert ourselves to-day, the strength of the destroyer will be shorn before the night comes. Do not throw upon the city authorities more than they can do. They have done and are doing all that any reasonable being can ask, but there is much more to do to-day than the city officials could do if their force were quadrupled.

We have referred to the fact that the people west, north, and south of Market, between Tenth and Twelfth, are comparatively healthy; those east and south-east of the pond, near Eleventh, are the exclusive victims. With the exception of the spot named, the entire city was almost free of cholera yesterday. We know of no case out of that locality. While thankful for this exemption, should not the exempted portions show their gratitude by laboring to-day in the work of purification? Let sanitary laws be forced on those who will not attend to them. Those who are well now owe this to themselves. Eternal vigilance is the price of health."

Every prediction made in this appeal was verified to the letter. Numbers of citizens went to the scene of disaster on Tuesday, and remained through the day, assisting in sanitary measures and in the removal of the inhabitants. In addition to these citizens, the authorities had a large force at work from Monday afternoon until the next Saturday. The benevolent feelings of relatives and friends induced some persons to remain in the infected quarter at night, and the attacks were confined to these. Of the multitudes who labored in the very focus of the pestilence, in day time, not one was attacked, thus showing that the basis of the foregoing appeal, contained in the declaration: "during the day, malaria has but little power; the night is the season of its strength," was a true one. If malaria is not the cause of cholera I know nothing whatever of the cause, and could give neither advice nor an opinion of its operations. The reader can judge whether this lamentable condition belongs to my conviction of the malarious origin of cholera. But it must be manifest that the full verification of the predictions of 1833, 1849, and 1850 made on various points of cholera, is positive proof that the habitudes of the cause of cholera are well known, or these predictions would have signally failed. The nature, the character and the elements of the remote cause of any disease whatever, are the same all over the earth, and they have the same laws all over the earth. The nature, elements, and character of small-pox contagion are identical in Timbuctoo, Archangel, Cairo, and New Orleans, and the same is true of the cause of any disease. The producing cause of chill and fever may be modified in its effects, by habits of life, and by various other means, but the mode of its formation is ever the same. And this is true of the cause of cholera—the elements of its formation, no matter where it may show itself, are ever the same, and those means that are successful in guarding against it in any one place, are equally successful in another. It would be a matter of public rejoicing, for it would be promotive of the public weal, if all medical men would inform themselves as well upon these subjects, as a part of the profession has done. All thoroughly informed medical men agree upon the use of preventive measures for guarding against cholera, and if all the profession were thoroughly acquainted with all the facts on the subject of cholera, and all the laws that govern its cause, there would be an incalculable gain to the world. And is not this the noblest mission of medicine? Is there anything in the science that more deserves the attention of members of the medical profession? A crop of weeds can be grown to order, if the seed are planted, but not with more certainty than the production of a cholera endemic, if its seed are fostered and nourished. A fire may easily be extinguished with water, but with no more certainty than cholera may be prevented by the proper means.

But what have we gained thus far in the facts already brought forward? Let us measure them. First, we have found that cholera can originate in a ship, just as any other disease can, and as ships have ever been notorious for originating disease within their own limits, there is ample reason for believing that the ships New York and Swanton originated the disease with which they were attacked in November, 1848. Neither the ships nor the passengers had been in the way of "catching" cholera, and this fact settles the non-contagious character of the malady. A disease that can be acquired without contagion is always acquired without it. Then again we have the additional evidence of non-contagion in the facts that the steerage passengers in the ship New York did not communicate the disease to any one of the cabin passengers of the vessel, nor to any of the crew, and though the steerage passengers scattered themselves over the city of New York the disease was not communicated. There was no local cause for it in New York, therefore the disease did not spread, and it ceased even among the steerage passengers of the New York, after they left the ship, thus showing that the cause of the disease was local, and the locality was on the ship. Prof. Dickson, of Charleston, S. C., once undertook to build up the theory of contagion upon the events that happened at Folly Island, in connection with the brig Amelia. But the careful reader will be astonished to find that there was not a case of cholera, except among persons who had been at the wreck, on which the cause of the disease existed. Several of the victims escaped through the quarantine, and went into the city, but no one, who was not at the wreck, took the disease. And from such truths as these Professor Dickson drew the extraordinary conclusion, that cholera was contagious! But to return: Although the Philadelphia Board of Health gravely declare that the coincidence of the attacks of the two ships at sea is shrouded in obscurity, and then undertake to clear it up by an imaginary cholera atmosphere in the neighborhood of Nova-Scotia, when we come to examine the matter thoroughly, we find the Philadelphia Board in the condition of those gentlemen who undertook to solve the problem proposed by Charles II, respecting the identity of the weight of a tub of water with, or without a fish in it—instead of the cause of cholera on board of the Swanton being shrouded in obscurity, and instead of a bewildering mystery in the coincidences referred to, it turns out that there was no cholera on board of the Swanton, until after her arrival at New Orleans. Her disease was dysentery. It would be much easier to prove that the Swanton caught cholera from New Orleans, than to show that the people of New Orleans caught it from her. There was cholera in New Orleans many days before the arrival of the Swanton; there was none on the ship until the day after she reached New Orleans. The facts, thus far then, indubitably show that cholera can originate locally.

Let us, however, fasten the point of the local origin of cholera more securely, for it is an exceedingly important matter. Year after year the idea that cholera first appeared in 1817 has been repeated in every imaginable form, and it is an error that has been prolific of no small amount of misery to the human family. Men write glibly about the travels of cholera from 1817 to the present time, and thus give rise to the idea that the cause of cholera is a traveller, instead of being of local origin. Abundance of proof shows that cholera dates much farther back than 1817, but I take the case of the ship Mangles, in 1815. Mr. Corbyn, an intelligent English surgeon, took passage in that ship, in 1815, for India, and at that time, stray waifs of cholera atmosphere lying out at sea watching for ships, had not been manufactured, for this was two years before Jessore started the disease upon the circumnavigation of the globe. Nevertheless, the ship Mangles, after being at sea two months, was visited with a terrible attack of cholera, and lost a number of her passengers and crew. Mr. Corbyn's testimony is indisputable, for he gave it in 1832, after a long intercourse with the cholera of India. Here then we find evidence of the local origin of cholera on this ship, in 1815. Again: Dr. Ayres, of Hull, England, whose intimacy with cholera in England, since 1832 is well known to the profession, *published in* 1818 *a description of a frightful endemic cholera that appeared in England, in* 1817, and he now declares that there was no difference between that endemic and the cases called Indian or Asiatic cholera. That was not imported, most assuredly. Dr. Thackerah, of Leeds, England, was a physician in a severe visitation of cholera in that city, in 1825, seven years previous to the reputed importation into Sunderland. The endemic of 1825 was not imported—it was local in its origin.

The second important fact to be gathered from Drs. Fenton and Buel is on the temperature connected with cholera. In New York, Dr. Buel testifies that a sharp frost arrested the cholera of 1848, in the fourth case. In New Orleans, about the same time, the weather was oppressively warm, and cholera raged with devastating power during the hot weather, and was arrested by frost, early in January. Here then we find that the cause of cholera is connected with temperature—it is the production of warm weather, and its power is arrested by cold weather. If its effects are displayed in winter, they are always exceedingly limited, in comparison with the summer effects, *and they show themselves early in the winter only when the incubation commenced in warm weather.* Numerous instances of this kind are familiar to readers of American medical histories. There is not one feature of the winter appearance of cholera, that has not often been seen in chills and fever, bilious remittent fever, plague, yellow fever, and other diseases of the same origin. A familiar fact, which speaks forcibly on this point, occurred in the history of Bethlehem, Connecticut. It should be well understood by those who have to look into the varying phases of disease.

In 1750 a severe form of fever attacked the town of Bethlehem, in the spring, immediately after drawing a body of water off from a woodland pasture. Ten years after this event, after a similar flooding in summer, and a draining off of the water, a frightful epidemic fever appeared in Bethleehm, in November, and raged in cold weather. There is no fact more familiar to well read medical men, than the appearance of diseases, in winter, whose cause belongs to hot weather, but a few examples may aid the purposes of this paper.

Dr. Davis, of Columbia, S. C., gives an account of a winter epidemic which commenced in that town in November, 1815, immediately after the common bilious fever. Dr. D. adds: "The most swampy situations, margins of rivers, and places most subject to the endemial autumnal bilious fevers, suffered most severely from the epidemic."

Dr. Withers, of Warrenton, Va., describes a winter epidemic in 1814 in Fauquier. He says: "The epidemic was most prevalent and fatal in *the marsh*, about Woodside's; the face of the country is low and very wet. There were some cases on Tinpot (run). These were the parts most severely affected by summer and autumnal disease."

In Winchester, Va., the autumnal epidemic of 1822 was followed by a winter epidemic. A snow storm in February covered the earth a foot deep. This, with cold north-east winds and rain in the last of that month and first of March, produced considerable sickness.

These are given merely as examples. They might be accumulated into immense masses, all proving that the winter epidemic is dependent upon the same cause as that which gives rise to the epidemics of hot weather. It is a very familiar fact to the profession that malarious diseases occasionally display great energy in cold weather, but no matter whether they show themselves in summer or winter, they exhibit themselves in malarious localities. The medical profession in this country never doubted the fact that an epidemic intermittent or remittent fever which prevailed in winter, originated from the same cause that produces summer and autumnal diseases, and why should the doubt arise respecting cholera? Since it is notorious that other forms of malarious disease often appear in winter, why should any one peril his reputation by saying that cholera is not malarious because it has occasionally been seen in winter? What public end is promoted by such puerilities? What public service is performed by such loose adventurers of false logic? The very fact that cholera has occasionally appeared in winter is a good reason for believing that it springs from malaria, because it conforms in that, to a well known and familiar feature of all the other diseases that arise from malaria.

Another prominent feature of malarious disease is found in the extraordinary length of the latent period, during which the remote cause may lie in the system without showing any of its effects. There is no other cause of endemic or epidemic disease, than malaria, which has this marked peculiarity. It is not unusual for persons from some of the New England States to acquire chills and fever during a visit to the West. They take medicine, disturb the disease by "breaking the chills,' as the process is called, and then they hurry off home to a region where such diseases are unknown. But in the course of a few months, three, six or nine months, the western acquisition reasserts possession, and the victim is seized with fever and ague again, in a region where such forms of disease never originate, and there are many such places in New England. This is an example of the latency of malaria. This latency is found in all malarious diseases. Oriental plague, yellow fever, bilious fever, dysentery, cholera morbus, &c. &c., are all diseases of hot weather, but occasional attacks of these diseases occur in winter, as may be easily determined by an examination of the works of Rush, Lind, Cleghorn, Russell, Cheyne and other observers. The reason of these peculiarities is that malaria may lie dormant in the system for weeks and months before it displays its effects. What is true of the diseases mentioned above, is true of cholera.

I have frequently referred in former papers on this subject, to the remarkable features of what was called the Walcheren disease. The fate of the splendid army sent out by England, to the island of Walcheren is familiar to the most of medical readers. The most vivacious attack of the imperial guard could not have mowed down the men with greater devastation than the malaria of the island of Walcheren did. Regiments, that landed in full strength and health, were soon reduced to twenty or thirty men. The sickness was wide-spread in its devastations, and the fatality was very great. The discovery was made, that it was futile to fight against the climate, and the broken regiments were returned to England. The Walcheren disease was peculiar in its singular effects upon the liver and spleen, and was easily recognized by its peculiarities, and it is recorded by the eminent physicians who were with the army at Walcheren, and returned with the troops to England, that no matter in what part of England the soldiers were stationed, no matter how good the health of the men might be on their return to their native land, cases of the Walcheren disease continued to manifest themselves for nine and ten months after the troops reached England, and no such disease was known among the inhabitants, who had not been to Walcheren, of those sections of Great Britain where the troops were quartered. This shows that the activity of the cause is not always in proportion to the malignity of the malady, for a more malignant intermittent never was known. Yet the cause was acquired in the Island of Walcheren, and was dormant for nine months, and in some cases, for a longer period. Again: an English Regiment was sent to India, and stationed in a region that was subject to yellow fever; at the proper season, that disease seized the regiment, and made such havoc that the healthy portion of the soldiers was transferred to a point where malarial disease was never known to originate. These troops went into garrison with a regiment that had been stationed for sometime at this healthy point, and among whom there was no sickness. Cases of yellow fever, however, continued to occur among the transferred soldiers, for four months, while there was not a case among the other soldiers occupying the same barracks. Here is a clear case of the latency sometimes exhibited by even as potent a cause of disease, as that which gives rise to yellow fever. And in strict accordance with these features of malarial disease, we find cases of cholera. In a district of Louisville, in which a fatal pestilence of cholera showed itself last summer, there were fifty cases and thirty deaths in the course of a few hours, on one side of a square, that was not by any means populous. There were persons who slept in the midst of this mortality on the night it commenced, and who fled from their lodgings the next day. Some of them went to the country, and remained in good health for one or two weeks, and were then fatally attacked with cholera, when there were no other cases within five miles of them. A notorious example in the winter of 1848, has been often quoted by the author of these remarks. I allude to the cases of Major Dix, of the United States Army, and two of his fellow travellers. Major Dix was at Memphis, where there was a number of sporadic cases of cholera, and after remaining at that city for sometime, he came to Louisville, which was free from anything resembling cholera. Major Dix remained in Louisville about a week, and passed on to Wheeling, apparently in perfect health. Between Wheeling and Brownsville he was seized with cholera, while in the mail stage, and died in a few hours. In the same stage, were two other passengers who had been at New Orleans a few weeks before, while the pestilence was carrying off its hundreds. One of these passengers reached Brownsville, where he was attacked with cholera and died in a few hours. The other reached Youngstown, and died with cholera. Although the stage was filled with passengers, the only persons affected with cholera were the three gentlemen who had been in a cholera atmosphere in the South, some weeks before. There was no such atmosphere where they died, because its existence can be known only by its effects, and there had been no other cases of the disease for months, in all that region of country, nor were there any others for months afterwards. These cases, then, show very conclusively that the cause of cholera can lie dormant in the human system for a considerable space of time. There are familiar examples where the hot weather, suitable for the formation of the cause of cholera, was speedily followed by cold weather, and cases of the disease were thus *developed* in a state of things that could not have *originated* cholera. If cholera is indifferent as to its seasons, why is it that ninety-nine in a hundred of its visitations occur in hot weather? and why is it, that all over the earth, where its limited attacks have been seen in winter, men have recorded that its range was entirly confined to those portions of cities that were notorious for malarial disease. This is recorded of Edinburgh, London, Paris, Moscow, Petersburgh, and of all other places.

Medical men do not regard the winter attacks of chills and fever, bilious fever, yellow fever, oriental plague, etc., as evidence against the malarial origin of those diseases. They know the winter visitations are as much due to malaria, as the summer devastations are, and why should cholera be the exception?

Another marked feature of malarious disease is that attacks of it occur more frequently in the night, than during the day. This is especially true of the violent forms of malarious disease. I have seen a great many visitations of cholera, and have closely observed endemics of it in the years 1832, 1833, 1849, and 1850, and in all, the marked predominance of night attacks was conspicuous. And the universal records of medicine show that this feature has been common to cholera, all over the world.

Malaria attacks persons in the lower stories of houses with more violence than in the upper rooms, except where the upper tenements are as filthy as rooms could be anywhere, as in Edinburgh, Glasgow, etc. I have seen this law of malaria in constant operation in all the cholera visitations I have ever seen, and it is a fixed fact that those who lodge in rooms elevated two and three stories from the ground are almost entirely exempt from cholera, while it is raging among those who lodge near the ground. Hosts of medical men have testified to the truth of the operation of this law in cholera, in all regions of the earth. And in further corroboration of the malarious features of cholera, take the testimony of Professor John Bell, who does not believe that there is such a thing as malaria. Yet his examination of the history of cholera induced him to bear the following decisive testimony:

"The chief home and seat of cholera is in low, damp situations—on the banks of rivers or near pools and ponds of water—or which are encumbered with vegetable remains or filth of any kind. Those parts of cities thus situated and circumstanced, have always suffered most, and sometimes have been the exclusive seats of the disease, in all the chief cities of Hindostan, Calcutta, Madras, Bombay, Seringapatam, etc.; of Russia, as in Moscow, St. Petersburgh, Astrachan; of Germany, as in Vienna, Breslau, Berlin, Hamburg; of France, as in Paris, and other places; of Great Britain and Ireland, as in London, Sunderland, Newcastle, Gateshead, Musselburgh, Dublin, Cork, etc.; this fact has been placed beyond a doubt."

I here republish my remarks made in the article from which I have already quoted freely.

But an objector urges that if malaria is the cause of cholera, we should have that disease every year. The objection does not amount to anything like reasoning. There are a great many of the well ascertained features of malaria, which we cannot account for; we do not know why quotidian fever prevails in one country, tertian in another, quartan in a third, and why remittent fever is the prevailing form in another. We know the fact that these varieties originate, prevail, and decline under a community of circumstances, that show a common cause for their production. If we find intermittent fever prevailing for a while, then a severe remittent type for a brief space, followed by a continued form, and as the latter declines, the remittent re-appears, and gives way to the intermittent form, we do not assume that new causes come into play. It is reasonable to say, that the same cause varies the degree of its power. It is remarkable, that this identical state of things is true of intermittent fever and cholera. It has often been recorded, that a severe form of intermittent fever began to prevail at some particular point, in the midst of which cholera appeared, and the intermittent disappeared, and as cholera declined, the intermittent re-appeared. This has been so often noticed, not only with intermittent fever, but with other forms of malarial disease, that we are on safe ground in saying that the same cause, varying in degree, produced the intermittent, or other malarial disease, and cholera. And as cholera is universally found under precisely such circumstances as we find other malarial diseases, obeying the identical laws that are universally recognised as belonging to that class of maladies, yielding in no instance to any other laws, it would be folly to look for any other cause for cholera, than that which is under the dominion of malarial law. To make the matter plain, let us take a single example. Medical men have often remarked, in describing cholera, that intermittent fever was prevailing in a special locality, when cholera appeared; they have recorded that the intermittent become more intense upon the approach of cholera. What does this mean, if it does not plainly say that the intermittents became more severe as the cause that produced them became stronger? In the midst of the operations of the cause producing severe intermittents, cholera appeared. Were there two separate causes of the diseases in the atmosphere at once? Where is the necessity for believing such an absurdity as is involved in that idea? But what is a grave intermittent? It consists of an intense cold stage unusually prolonged; re-action comes on slowly and feebly, and the sweating stage is generally absent. The most intense intermittents have no re-action, and in them there is the most perfect identity of symptoms with those of cholera. No man could tell one disease from the other. Senac, an accomplished physician of the times of Louis XV., describes a malignant intermittent in such terms that his description of it could be applied to cholera without making any alteration. No physician can read Senac's graphic account of the symptoms of malignant intermittent without being forcibly struck with the similarity of the symptoms to those of cholera. We know of no other cause than malaria that can produce those symptoms uniformly, nor can any one name another. Why seek for another when we have one equal to the production of all the effects, especially when we know the laws that rule that cause? If then, malaria can produce such identical symptoms in malignant intermittent as are found in cholera, why seek two causes for the same effects? That which is capable of producing ten cases of malignant chill, can produce ten thousand, if it is extended in its influence.

The principles of the Newtonian philosophy are universally regarded as the basis of truth, and when they are rigidly applied to medical subjects, there will be more

5

exactness in their principles. The first law of Newton's philosophy, is: "We are to admit no more causes of natural things than such as are both true and sufficient to explain their appearances." Rule second—"To the same natural effects we must, as far as possible, assign the same causes." Rule fourth—"In experimental philosophy, we are to look upon propositions collected by general induction from phenomena as accurately, or very nearly true, notwithstanding any contrary hypothesis that may be imagined, till such time as other phenomena occur, by which they may either be made more accurate, or liable to exceptions." These are the laws upon which all modern philosophy stands, and upon their secure basis, we confidently trust the malarial origin of cholera.

But why does not cholera appear more frequently? We might as well be asked why intermittent, remittent, and bilious fever, dysentery, cholera morbus, etc., do not come, without the conditions necessary to produce them; but as many regard the question propounded above, as a very reasonable one, we shall proceed to answer it. The varying phases of malarious disease are as dependent on the varying conditions of seasons, as the productions of the soil are. Those climates that are very uniform in their harvests, are uniform in the character of their diseases, and no violent departure from their customary health can take place without a noticeable change in their meteorology. Thus we find the epidemics of the middle ages ushered in by tornadoes and floods of rain; they expended their energies first upon the crowded, filthy, and wretched quarters of the poor, aud became general in a city where the general atmosphere was contaminated by a persistence of the conditions that had originated the cause in the suburbs, the filthy lanes, closes, etc. This has been the record of cholera, too, all over the earth. An attentive examination of the facts of physical geography gives clear and satisfactory reasons for all those phenomena in cholera that have been looked upon as remarkably mysterious. Those who have derived their notions of physical geography from Mercator's projection, have been surprised with what they call the erratic course of cholera; its departure from straight or direct lines, its appearance at a particular locality, after having already shown itself at a point far beyond it. All these things are in strict accordance with the isothermal lines of Humboldt, and any one familiar with these lines, and with the doctrines of malaria, would have looked for those very features, that so long bewildered mankind. There is quite as much mystery in the departure of vegetation from a straight line, in the northern zones, as there is in the wanderings of cholera. If, for example, we expect vegetation to follow the parallel lines of a map, under the idea that the distribution of heat is in parallel lines, we find a mystery at once in Lapland. The angular projection of the part of that country which produces barley and other food, for human beings, is a matter of surprise until it is found that the isothermal line makes a similar angle. There is a fact of great importance that should be kept in view; in torrid zones the isothermal lines are very regular, and diseases of seasons are equally so, (cholera swept over 10,000 miles there sooner than it did hundreds in the temperate zones;) where those lines are erratic, diseases, that are dependent upon climate, are also erratic. The law, as expressed by physical geography, on the subject of plants, is true of malarial diseases. That law is: "lines of equal heat do not follow the parallel of the equator, but have convex and concave summits, which are distributed very regularly over the globe, forming different systems along the eastern and western sides of the old and new world, in the centres of continents, and in the vicinity of the ocean. Temperature determines the habitation of races, and consequently, the extension of the vegetable kingdom, the forms of plants, in general presenting the same relations under the same isotherms; and as these lines form curves at unequal distances from the equator at different points in their course, so the vegetable zones, determined by the degree of their temperature, follow the same inflections." This law applies as perfectly to malarial disease as to plants. The operation of extreme temperatures modifies the growth of plants, and the production of malarial disease. It is impossible to fix with certainty anything that varies so much as temperature does. What Physical Geography calls isothermal lines, isocheimal and isotheral curves are only an approximation to a general result. A particular locality may have for a series of years almost a uniform annual mean temperature; a departure may take place from this uniformity, and excessive heat and a great deal of rain may present themselves. Just as certainly as this departure takes place, there will be a corresponding change from the health of that locality; and in this way, a place that may have acquired a great reputation for health, may be made the seat of a dreadful pestilence; a striking illustration of which shall be given directly. Another law of Physical Geography must be understood in order to understand the philosophy of malarial endemics. The annual mean temperature of the earth is about the same, year after year. It follows then that if any particular locality has an unusual amount of summer heat, in any particular season, some neighboring point must have a corresponding diminution; and if other circumstances be favorable, the point that has the usual amount of heat, will have an unusual amount of sickness, while the place that has the diminution is healthy, even if coterminous with the seat of pestilence. We find many striking illustrations of these facts, but there is one here at home, that is so perfectly to the point, that we prefer its use, although we have frequently referred to it, in other publications. In Lexington, Ky., in 1833, there was an unusual amount of summer heat, attended with an extraordinary quantity of rain. Portions of the town are remarkably well drained, while other parts are not in a condition to be drained, when rains are excessive. In a portion of the flat part of Lexington there was a deposit of the scrapings of the streets, and of the filth of the city for many years, and upon this mass of dead vegetable matter, the unusual heat, and the floods of the spring poured down their power. Here then were all the elements for a malarial pestilence, and it assumed the form of cholera. The disease commenced at the filthy spot we have mentioned; it raged with peculiar fury in all that part of the city that was under the influence of the locality we have named, and ravaged other parts in a ratio corresponding to their approach to similarity of condition. Large portions of the city were exempt from the pestilence. We visited Lexington some weeks after the cessation of cholera, and with the assistance of a gentleman perfectly familiar with the history of the pestilence, we made a map of the place, on which was indicated all the high and flat parts of the town, and the number of deaths in each house. In analyzing that map, we were amazed at the tenacity with which cholera clung to the flat and undrained localities, for where the cause of the pestilenre exhibited the remarkable power it did in Lexington, one might have reasonably supposed that the deleterious atmosphere would have travelled more frequently than it did, beyond the exact source of its production.

During the rage of the pestilence at Lexington, Versailles, only twelve miles distant, enjoyed good health. Instead of an unusual summer heat at that place, there was a diminution; and there was nothing like an excessive season of rainy weather. But in 1835 these conditions were reversed. Lexington and Versailles both had a good deal of rain, so much indeed that the people of Lexington dreaded another visitation of pestilence, and the crops of

Woodford county, of which Versailles is the county-seat, were much injured by the rain. But, though the rain was abundant in both places, there was a remarkable difference in temperature. While the heat was severe in Versailles, the people of Lexington were obliged to have fires in their houses in the evening to keep themselves comfortable. That was the year of pestilence in Versailles, and Lexington escaped. These are potent examples of the truths we have been endeavoring to inculcate. These are forcible truths upon a momentous subject, but I am anxious to make the subject as clear and convincing as possible. Look to the facts now in Louisville.

In this city, in the years 1832, '33, and '49, cholera commenced each year in the same square, and within a few yards of the same spot. In 1849, this spot was cleansed, and thorough work was made of the cleansing. The result was, that in 1850, the worst cholera year that Louisville has known, there was not a case in that ancient haunt of the malady. In 1833, a number of localities was visited by cholera; in 1850, some of these places had undergone great changes; they were thoroughly drained and improved with good dwellings. In these improved spots there was not a case of cholera in 1850. But in those spots that continued in 1850, in the condition they were in in 1833, cholera was severe. What stronger proof could we have of the local origin of the disease, even if those facts stood alone? Again: In 1850 a green pool of water stood in a vacant lot fronting on Jackson street, in Louisville, and run behind some four of five houses on the south side of Jefferson street. There was not a house on either side of Jefferson street, extending as far as the length of the pool already mentioned, that was not attacked by cholera in one night. Those who lived south of the pool, much nigher to it than the victims on Jefferson street, escaped an attack. The south wind blew the malaria from them upon the people on Jefferson street, and the range of the cholera on Jefferson was precisely the width of the length of the pool of marshy water. Those who lived above or below the length of the pool escaped, though living otherwise in as favorable a condition for cholera as those who were attacked by it.

On Market street, between Eleventh and Twelfth streets, opposite the lower market house, a number of people lived in a flat, undrained locality. Pools of water stood under some of the houses; the cellars had been full of water repeatedly, and some of the lots and houses were very filthy. A short distance from this square stood an old pond, in the edge of which large quantities of hemp-shives had been thrown, and the evaporation of the water had brought a considerable mass of this putrid matter to the surface. The evening wind blew across the pond, and carried malaria to those who had an abundance of it in their dwellings. A frightful cholera commenced in the night, and by 10 o'clock of the next night, in that sparsely populated street, there were fifty cases of cholera, of which thirty died in a short time. There were about one hundred deaths in all. The disease reached as high as the third house above Tenth street, and was restricted to the locality where it originated. As soon as the endemic showed itself, active measures were resorted to, for the removal of the cause; the people were removed, the edges of the pond covered with lime and sand, and the dwellings purified with lime. In this way the disease was restrained within the limits of its outbreak, and did not extend beyond the points of its first development. And there is a fact that should be noted, both as evidence of the malarious origin of the disease, and as a source of confidence to others hereafter. On the night of the commencement of the disease in the square we have described, the writer of this essay, fearing the evils of consternation when the people should hear next morning of the night's dreadful work, drew up an appeal urging the public to go to the assistance of the afflicted. The people were assured that no one would run any risk in day-time, but those who remained at night would suffer. In response to this appeal, great numbers went down, and worked hard for a number of days. Of these not one suffered. But several of those who nursed at night took the disease and died, the two sets thus vindicating the truth of the prediction in the published appeal I have quoted. Malaria is almost innoxious in day-time, but is fatal at night; it, therefore, was the cause of the fearful pestilence that raged in the infected section of Market street.

Of the people removed to healthy parts of the city, many escaped an attack of the disease, but a number were assailed by it in various parts of the city, and some in the country. They died among the friends who had sheltered them, but those friends escaped in every instance.

These facts clearly establish the local origin of cholera. But for the local circumstances which we have shown are present all over the world wherever cholera has manifested itself, there was no reason why the localities we have described in Louisville should have had cholera in preference to other squares. And in full corroboration of the local origin of cholera, the London Gazette for August, 1850, makes the following remarks:—

"*The tendency of Cholera to revisit the same houses and streets.*—As was anticipated and predicted, cholera, during its recent visitation, returned to the same countries, and the same cities and towns, and even the same streets, houses, and rooms which it ravaged in 1832. It is true that many places have been attacked in the recent which escaped in the former epidemic; but very few indeed that suffered then have escaped now. In some instances, it has reappeared on the very spot in which it broke out sixteen years ago. The first case that occurred in the town of Leith in 1848, took place in the same house, and within a few feet of the very spot whence the epidemic of

1832 commenced its course. On its reappearance in the town of Pollokshaws, it snatched its first victim from the same room and the very bed in which it broke out in 1832. Its first apppearance in Bermondsey was close to the same ditch in which the earliest fatal cases occurred in 1832. This return to its former haunts has been observed in several other places, and the experience abroad has been similar. At Groningen, in Holland, the disease in 1832 attacked in the better part of the city only two houses, and the epidemic broke out in these two identical houses in the visitation of 1848."

Cholera is, therefore, no traveller; it is a denizen of the place, wherever it appears, and is indigenous to the soil. If it does not come every year, the reason may be found in the phenomena of failure of crops. There is no more reasonableness in supposing that cholera should come every year, if we look upon it as a malarious disease, than there would be in looking for a good fruit year annually, or a successful yield of the harvest every season. What seem to be very trivial changes in seasons, have a marked effect upon crops, and they also effect the public health. One state of natural phenomena produces intermittent fever; a different condition does not produce it, and the same is true of the great disease of the times—cholera.

It may be doubted whether in full view of all the circumstances connected with cholera, the pestilence has been a great evil to the world. If it has slain its thousands, it has taught and impressed truths that will be the salvation of myriads in the future progress of years. Governments and people have been taught the value of cleanliness, of ventilation, of pure air, and the imminent and certain danger of overcrowding. These things should have been known and valued at all times, but cholera seems to have roused the knowledge into great activity. When diseases are curable, people lean upon the doctor; but when, as in cholera, there seems to be no cure in the generality of cases, the minds of men must be turned to the means of prevention.

In 1834, in a prize essay on Quarantines and Sanitary Regulations, written by Professor Caldwell, far in advance then of what sound and correct views of the habitudes of cholera have done within the past few years, that eloquent and able teacher of the laws of malaria, uttered the following prediction: "Cholera, though a fatal scourge to the world, will through the wise and beneficent dispensation under which we live, be productive of consequences favorable alike to science and humanity. Besides being instrumental in throwing much light on the practice of physic, it will prove highly influential in extinguishing the belief in pestilential contagion, and bringing into disrepute the quarantine and sanitary establishments, that have hitherto existed." How remarkably all this has been verified, is well known to the medical reader.

I think that the truths presented in this paper are ample for the proof that the cause of cholera is subject to those laws that belong alone to malaria. There is not a law of malaria that cholera does not implicitly obey, and the measures of sanitary regulation that control malaria have universally controlled cholera effectually. The recent records of English medicine are full to overflowing, on this important point, and fully substantiate it. And in view of these truths, we cannot but look upon all attemps to call public attention away from truth piled upon truth, to the miserable chimeras and fancies of ignorance, as deleterious in whatever degree these last mentioned influence the public mind. I must pay a passing tribute to a recent example of this kind.

In the February number of the Western Lancet, a Mr. Lea, a geologist, has undertaken to enlighten the medical profession upon the cause of cholera, and in his plastic hands, the problem is resolvable into geological formations and solutions. Mr. Lea belongs to the Wernerian or aqueous school, and is amazingly generous to leave the feuds of his own science, to mingle in those of which he knows nothing. What has geological study taught him on the etiology of disease? What acquaintance with the operations of the remote, predisposing, proximate, and exciting causes of disease has he obtained from the study of Trilobites, Ammonites, Pterodactyls, Plesiosaurian and Iguanodon reptiles? With a wonderful ignorance of medical literature, Mr. Lea complacently announces: "the progress of investigation and experience, (fortunately for the interest of humanity) leads to the confirmation of all that has been published, respecting the truth of the so called geological theory of cholera. All other theories in this connection, have had an ephemeral existence, and fade away before the lights of science and experience." In a spirit of charity I must ascribe Mr. Lea's statements to superlative ignorance, for on any other plea, his language would exhibit a spirit of untruth that would approach the atrocious. The geological theory never has obtained any credence among medical men, and has scarcely had any serious consideration. Take an example of the ridiculous absurdity of the geological notions. M. Tardieu says: "M. Boubée communicated to the Academy of Sciences his extensive researches upon the progress of cholera, compared with the geological composition of the places marked by its passage. It resulted from this study that the ancient formations were spared, while the tertian and alluvial strata suffered most. M. Boubée concluded that in France the points at which one would most certaily escape the scourge, ought to be the greater portion of Bretagne, Limousin, Auvergne, Cevennes and the Pyrenees. But to show how little credit should be attached to such assertions, it is sufficient to add, that among the places designated by geology as the most fatal, we find Lyons, Guyenne, and Gascogny, which in fact were wholly spared." And M. Littré and M. Monneret say: "all that has been written to prove that the cholera is more prone to attack those soils which are identical in geological constitution is in manifest opposition to numerous contradictory facts." And now this miserable Harlequin, thus scouted and scourged by science, has the impudence to blow its penny whistle and not only to command the world to listen to its syren notes, but to swear there is no other music in the universe!

As to the ephemeral medical theories of which the geological Mr. Lea speaks, we beg leave to refer to his careful perusal, the great work recently published by Dr. C. P. Riecke, a distinguished Prussian physician. The author makes a minute survey of cholera, and incontestably establishes the following truths. Dr. Riecke says: "Cholera is a malarious disease—a sort of malarious neuralgia, which acts through the vitiated state of the humors brought on by miasmata. Cholera is to be classed in the same category with ague, typhus, the plague and yellow fever. The effects of effluvia are as conspicuous with cholera, as with the yellow fever and the plague." The London Lancet speaks in warm terms of commendation of Dr. Riecke's valuable work, and the January No. of the British and Foreign Medico-Chirurgical Review thus speaks of it: "the work of Dr. Riecke contains much important matter as to the localization of the disease in Berlin; it is altogether a very valuable contribution to sanitary science." And this work is devoted to the malarious origin of cholera, one of Mr. Lea's ephemera. And the labors of such men as Drs. Guy, Grainger, Frost, Rogers, Sutherland, Lewis, Thorn, Baly, Gavin, Bushnan, all the Sanitary Boards of England, composed of men who understand sanitary science, and who base all their regulations upon means that cause the prevention of malaria, are to go for nothing, in the presence of Mr. Lea's farthing light! He drives at such men with as much nonchalance as he would chip an intrusive piece of carbonate of lime from a Pentacrinus Briareus. While Great Britain is in possession of such laborers as those who are adorning her sanitary science with substantial honors, we do not think it marvellous that the English Government did not vote Mr. Lea a gold medal for the suggestion he made to the British empire, by letter, to prevent the appearance of cholera by the use of rain water!

Mr. Lea very confidently announces the following statement: "it will be found that all those extensive regions of primary and arenaceous formations, where no calcareo-magnesian water is used, have again escaped the much dreaded epidemic, as they did heretofore; and that no *well attested fatal* case occurred among those who used *rain water exclusively ;* and I may add *boiled* water, though it is of less purity than rain water, and therefore not so entirely to be relied on when taken alone."

Now it is notorious to medical readers that the only known place which has been afflicted with cholera every year since 1818, is precisely that kind of a spot, or formation, which in Mr. Lea's geological judgment is a certain safeguard against cholera. About 260 miles N. W. of Madras, "a rounded mass of rock towers up abruptly from the plain. *This is not one of those limestone rocks common in some parts of India, which are formed by the evaporation of water deeply charged with calcareous salts. The rock is granite;* about half a mile in diameter, and some 500 feet high." Yet, on this granite or primary formation, where no calcareo-magnesian water is used, cholera annually asserts a sway which it has not exercised in any calcareo-magnesian deposit on the earth. The British and Foreign Medico-Chirurgical Review says: "the place of burial of each successive regiment bears sad testimony to its (cholera's) permanent and unrelaxed activity." Here then is a granitic rock, 500 feet above the plain, with no calcarao-magnesian water in the neighborhood, with every geological requirement for salubrity, proven by the annual records of thirty years, the most permanent and fatal spot in the world for cholera visitations. If it is possible by any means to let out all the inflated consequence of any absurdity, how could voluminous tomes more effectually destroy anything, than this fact destroys Mr. Lea's geological phantasy. Mr. McGregor and Mr. Ferrier, medical officers at Bellary, indicate sources of malaria, but the calcareo-magnesian solutions, essential to Mr. Lea's rickety crotchet, cannot be found within many miles of the Bazaar of Bellary. And it must be remembered by Mr. Lea, while inflating his geological puff-ball with the statement that, "all those extensive regions of primary and arenaceous formations, where no calcareo-magnesian water is used have again escaped," that there are also extensive regions where no primary rocks are found, where the most modern groups are to be found, and in the most perfect regions of the Poikilitic system, where calcareo-magnesian water is exclusively used, in which there has never yet been a case of cholera. There is no difference between the "formation" and the drinking water of those portions of Louisville where cholera prevailed, and those extensive portions of the city in which there has never been a case yet. Cholera commenced in Louisville in 1832, 1833, and 1849 in the same spot; in 1850 this place was thoroughly cleansed, and in that year, when there was an unprecedented amount of cholera in Louisville, there was not a case in its former haunt. Parts of the city that had cholera in 1832, and 1833, and which remained unimproved, had it in 1850, while portions in which the cholera was severe in 1832 and 1833, which have been drained, cleansed, and improved with good buildings, had no cholera in 1850, though some of the other portions referred to were contiguous to them. But the geological formation, and the drinking water, in all these various aspects of the pestilence remained unchanged.

Mr. Lea may peck away at these facts with his geological hammer as much as he pleases, but he cannot recover his theory from its submerged condition.

It is needless to waste much time on such a notion as Mr. Lea advocates in the Western Lancet, and its appearance in that periodical is my only apology for even noticing it. There is the less reason for much notice, from the fact that Mr. Lea seems to be perfectly indifferent as to whether what he says is true or not. For instance, Mr. Lea asserts: "the plantations on the Mississippi, *now* supplied with rain water, and the towns and villages supplied with the same, *all escaped*." Mr. Lea lays stress upon the word *now*, as though the introduction of the use of rain water, as a drink, in Mississippi were quite a recent custom, but there is no approach to truth in this, or any other part of his statement. Cholera prevailed in Mississippi plantations, where rain water alone was used, just as it did on those where it was not used; and those who used river water fared quite as badly as those who used calcareo-magnesian water. If Mr. Lea had investigated the subject with an eye to truth, or for any other purpose than that of nourishing his miserable little bantling, he could have found on the plantations of Col. Player, Mr. Maher, Mr. Morancey, at Millengen's Bend, and all through the plantations on Walnut Bayou, how little the use of rain water has to do with the prevention of cholera. The cholera was as virulent, as extensive, and as fatal on plantations where the purest rain water only was used, as where well water was the sole article for drink and culinary purposes.

But to mark, beyond all possibility of mistake the character of Mr. Lea's offspring, take two examples. Ledbury, in Herefordshire, England, stands on the blue limestone, with the anticlinal axis, just as Cincinnati does. The town stands on the summit of the rock, and of course is dependent on water that percolates through the limestone strata. This water is exceedingly "hard," being more saturated with calcareo-magnesian salts than the well water at Cincinnati. *There never has been a case of cholera in that town,* but according to Mr. Lea it should be there day and night.

Take another. The village of Fochebar, Rosshire, Scotland, stands upon the old red stone, which Hugh Miller has made famous. The sand stone rests on gneiss, at the back part of the village the gneiss crops out. This is primitive rock, and there is no limestone, nor magnesian formations within fifty miles of the place. The water is as "soft" as the purest snow water. Here then there should have been no cholera, according to Mr. Lea, yet stubborn history says that one visitation of that disease swept away every human being, except one old woman. And numerous villages in Scotland, on the same geological "formation" were dreadfully afflicted by cholera.

How pitiable it is for a man, ignorant as he can be of a grave and important public question, involving the lives and happiness of the whole human race, to attempt to draw away the minds of people from the true sources of disease, and to fix them upon one that has no kind of connection with the cause, and which rests on no better foundation than the whimsies of a distempered imagination! All the wealth of California could not induce me to utter a statement on the cause of pestilence, that I do not *know* to be true, and fully entitled to public confidence. I know the responsible duties of public hygiene, and when I cease to be true to them, let my name be execrated.

But to sum up all in a few words: the geological conjecture does not and cannot account for the fact that cholera prevails in hot weather, and is checked or controlled by cold; it cannot explain why it hangs upon the borders of rivers, near marshes, in damp and filthy localities; why it follows the direction of a wind, and destroys seven hundred persons in one quarter of a town, as Augusta,

Ga., while there is not a case in all the rest of the city; why it attacks one side of the street very fatally where there is not a case on the opposite side; why it is fatal among sleepers in the lower stories, and not in the upper stories; and why it does not prevail every day in the year. There is not one feature of cholera explained by the geological theory, and it is unworthy of any attention, especially when it is completely ignored by the only profession capable of passing a judgment on its demerits.

Upon one more statement of Mr. Lea, I must be permitted to make a remark. He says: "last summer, when the cholera broke out in Louisville, it was reported to be caused by the water of a well being poisoned." A more senseless report never was started. Medical men know that many persons may be exposed to malaria, and that a number will escape its influence; toxicologists know that if a poisonous water, capable of destroying life, be taken into the stomachs of one hundred persons, every one will show the effects. Numbers of persons in the houses in which there was the greatest fatality, who used water from the same pump used by those who died, were not even sick. The pump handle was removed, but abstinence from the water did not stop the disease. Persons who left the district, and went to parts of the city in which there was no cholera, succumbed to the disease a week after having quit the use of the fatal water! They remained perfectly well up to the onset of cholera. This was the case with a number of persons; in the localities in which they died, there had been no cholera, nor has there been since. In addition to these conclusive facts, there were families among the first victims who did not use the water of the suspected pump. But Mr. Lea's pump story is quite as rational and useful as the rest of his whimsical notions.

Dr. Starr, of Cincinnati, has recently published a paper, entitled, "Reasons for concluding that an excess of carbonic acid is the atmospheric cause of cholera." This is a very respectable paper, and the statements are fairly presented. But there are fatal objections to the theory. For instance: a concentrated carbonic acid gas, capable of producing the overwhelming effects that are often seen in cholera, as for instance on the 24th of July, in Louisville, would not produce the symptoms of cholera. Cholera does not commence with "a sensation of great weight in the head, giddiness, a sense of constriction in the temporal regions, a ringing in the ears, with a pungent sensation in the nose, a strong tendency to sleep, accompanied by vertigo, and so great a loss of muscular power that, if the individual be at the time in an erect posture, he instantly falls, as if struck to the ground." These are symptoms of the poison of carbonic acid gas, when it is sufficiently concentrated to destroy life, but these are not in any degree the symptoms of cholera. A man cannot imbibe a fatal dose of carbonic acid gas, and carry it two or three weeks before any effect of the dose is seen. Dr. Starr should have suspected his views, when he had to imagine that "there was," in the track of the packet New York, in the latitude of Nova Scotia, "an ocean bed of carbonate of lime, yielding its carbonic acid under the chemical influences exerted by the decomposing agents of the ocean." This is drawing the matter rather strong for philosophical medicine. There is not one oceanic fact to justify the notion. One of the laws of carbonic acid gas is that it is rapidly absorbed by water, and it cannot be let loose at the bottom of the ocean, and sent up from the depths of the sea into an emigrant ship.

Dr. Starr should have remembered too, while accounting for cholera, in Russia, in winter, that according to his views cholera should be a winter disease more commonly than it is. I am aware of the fact that he states there is less carbonic acid made in winter, on account of the arrest of decomposition by a low temperature. But this does not meet the difficulty, as Dr. Starr himself finds, when he undertakes to account for winter cholera, in Moscow. He there finds that abundance of the gas is manufactured in hot, close, unventilated subterranean rooms. And from what is known of the desolating effects of cholera, from overcrowding human beings in small apartments, in summer, when ventilation is used to some extent, what should be the case in winter when houses are made as tight as possible to keep out cold weather; when fires consume the oxygen of the room, and carbonic acid gas is extensively made by the lungs, even in a low temperature? Cholera should constantly appear in winter, under these circumstances, and overcrowding should be more fatal in winter, than in summer. But truth gives a different result.

I hope that Dr. Starr will pursue his investigations. He has moved so far on the road to truth, that I am persuaded the use of facts, logic, rigid induction and analysis will lead him in the paths of safety.

I am wedded to the malarial origin of cholera, not for the sake of the theory, but because it is the only view that explains all the phenomena. *There is not one law of malaria that is not found in the habitudes of cholera, nor is there a feature of cholera that is not confined within the circle of the powers of malaria.* There is not one useful sanitary regulation in use in any part of the world, that is not in strict conformity to the doctrine of malaria, and this is a testimony to its truth, the more valuable from the fact, that the sanitary laws are often used by those who repudiate the malarious origin of the disease. It shows that there are others besides Moliere's Bourgeoise *gentilhomme*, who may talk prose without knowing it.

I regret that my space is exhausted for the present. Much the most interesting part of my undertaking remains to be done; and that is the drawing up of a record of the triumphs of medical hygiene, over the great pestilence of the times. This must be deferred, however, until another month, and I hope in my next to show how earnestly, zealously and effectually, the medical profession have labored in the service of humanity. In no one thing has medical philosophy more completely entitled itself to the gratitude of mankind than in its victories over the cause of cholera, and those triumphs I shall endeavor to record hereafter.

