# Dr. E. D. Fenner

**Published:** 1851-06

**Authors:** 


### DR. E. D. FENNER.

We regret very much that we fell into the singular error, in our April number, of calling Dr. Fenner, Editor of Southern Medical Reports, Dr. Fenton. In the essay on "Epidemic Influences," Dr. Fenner is invariably called Dr. Fenton. We know how the mistake was made, but we cannot see how it escaped detection for the length of time it passed unnoticed. We are consoled, however, with the reflection that it is moɹe easy to rob Dr. Fenner of his name, than of his reputation.

